# Modeling neuronal dynamics during brain ischemia

**DOI:** 10.1186/1471-2202-12-S1-P354

**Published:** 2011-07-18

**Authors:** Bas-Jan Zandt, Bennie ten Haken, Michel JAM van Putten

**Affiliations:** 1 Department of Applied Physics, University of Twente, Enschede, The Netherlands; 2 MIRA-Institute for Biomedical Technology and Technical Medicine, University of Twente, Enschede, The Netherlands; 3 Department of Clinical Neurophysiology, Medisch Spectrum Twente, Enschede, The Netherlands

## 

Neurons are critically dependent on sufficient supply of oxygen and glucose When this supply is subcritical, as in ischemia, the signaling processes of the neurons are altered due to synaptic transmission failure [1] and decreased rates of the molecular ion pumps needed to maintain transmembrane ion gradients [2]. These changes are partially reflected in the electroencephalogram (EEG), as this is a sensitive measure for global network function, reflecting extracellular currents, mainly from synchronous activity of pyramidal cells [3]. Modeling a neural network suffering from ischemia may further improve our understanding of the effects of ischemia and the relation with the EEG. This may contribute to improved methods for diagnosing pathologies related to ischemia and hypoxia.

The electrophysiological behavior of a single neuron, which we calculate with a Hodgkin-Huxley type model, depends heavily on the intra- and extracellular ion concentrations. These are usually assumed to be constant, because the molecular ion pumps (ATPases) of the neurons and glia maintain the concentration differences needed for the generation of action potentials. During ischemia however, the required energy (ATP) for the pumping process is not fully supplied anymore and these concentrations change. This is calculated by integrating the average fluxes of the individual ion species across the cell membranes, following an approach similar to those used by Dronne [4] and Cressman [5].

The pump rates are assumed to be linear with the total energy use of a cell, for which Michaelis-Menten kinetics depending on the oxygen concentration are assumed. These concentrations depend on the position of the cell relative to the blood vessels.

With a model for the behavior of a single neuron during hypoxia, the behavior of a network of these neurons, perhaps with different properties, and the resulting EEG signals can be simulated.

We will present our first results of the influence on the electrical activity of the oxygen supply to the cells in the network.
